# Overexpression of Pea DNA Helicase 45 (*PDH45*) imparts tolerance to multiple abiotic stresses in chili (*Capsicum annuum* L.)

**DOI:** 10.1038/s41598-017-02589-0

**Published:** 2017-06-05

**Authors:** Tagginahalli N. Shivakumara, Rohini Sreevathsa, Prasanta K. Dash, M. S. Sheshshayee, Pradeep K. Papolu, Uma Rao, Narendra Tuteja, M. UdayaKumar

**Affiliations:** 1 Department of Crop Physiology, University of Agricultural Sciences, GKVK Bangalore, India; 2 ICAR–Indian Agricultural Research Institute, Pusa Campus, New Delhi, India; 3 ICAR-National Research Centre on Plant Biotechnology, Pusa Campus, New Delhi, India; 4 Amity Institute of Microbial Technology, Amity University, Noida, Uttar Pradesh India

## Abstract

Imparting tolerance to abiotic stresses is of global importance as they inflict significant yield losses in field as well as in vegetable crops. Transcriptional activators, including helicases are identified to play a pivotal role in stress mitigation. Helicases, also known as molecular motors, are involved in myriad cellular processes that impart intrinsic tolerance to abiotic stresses in plants. Our study demonstrates the potential of a Pea DNA Helicase 45 (*PDH45*), in combating multiple abiotic stresses in chili. We harnessed *Agrobacterium*-mediated *in planta* transformation strategy for the generation of stable, single copy transgenic events. Precise molecular detection of the transgenes by sqRT-PCR coupled with genomic Southern analysis revealed variation in the integration of *PDH45* at distinct loci in independent transgenic events. Characterization of five promising transgenic events showed both improved response to an array of simulated abiotic stresses and enhanced expression of several stress-responsive genes. While survival and recovery of transgenic events were significantly higher under gradual moisture stress conditions, under imposition of moderate stress, the transgenic events exhibited invigorated growth and productivity with concomitant improvement in water use efficiency (WUE). Thus, our study, unequivocally demonstrated the cardinal role of *PDH45* in alleviating multiple abiotic stresses in chili.

## Introduction

Plants, being an integral component of the soil–plant–atmosphere continuum (SPAC), are directly affected by the abundance/scarcity of environmental factors such as water, salt and temperature. These three factors, generally grouped as abiotic stresses, globally inflict 70% of yield loss^1^. Various plant processes starting from seed germination to maturity are physiologically and biochemically affected through discrete molecular responses to these abiotic stresses^2^. Thus, improving abiotic stress tolerance in crops is imminent to cater to the needs of increasing population under dwindling agricultural land and natural resources especially under the anthropogenic global climate scenario^3^.

Dissection of genetic architecture of abiotic stress tolerance has revealed it to be a complex polygenic trait^4^. Plants have evolved diverse adaptive strategies to cope with water-deficit conditions and these mechanisms are often classified under drought avoidance, drought escape, and drought tolerance strategies. Recent opinions on drought adaptation strategies have emphasized the identification of specific traits associated with water mining, water use efficiency (WUE), and water conservation traits. While these traits are constitutive and integral, several cellular level tolerance (CLT) mechanisms have been identified as acquired tolerance traits^5^. Under water-deficit conditions, CLT mechanisms, which bring about alterations in cellular metabolism for the plant adaptation assumes significance. Lately, plant biotechnology has emerged as a driving force for crop improvement. Identification of a plethora of superior genes and introgression of these genes into plants has demonstrated that the technology has the propensity to develop improved crop varieties that can mitigate multiple stresses^6^. There has been increasing research efforts to identify the genes, which modulate the processes of CLT. These mechanisms involve scavenging reactive oxygen species, osmotic homeostasis^7^, membrane/protein stability and maintenance of protein turnover^8^. Translation, protein turnover and protein stability are most critical under stress. Stress induced decrease in translation could be due to formation of secondary structures in the translation initiation site, leading to the production of truncated non-functional proteins^9^. Therefore, use of stress responsive genes that control or avoid the formation of secondary structures form an exciting approach to develop stress tolerant plants.

Among the array of genes deployed for abiotic stress tolerance, “Helicases” have been elucidated as major and effective players in alleviating multiple abiotic stresses^10^. In plants, DNA helicases function as molecular motor proteins in various cellular mechanisms and are essential for almost all DNA metabolic activities including pre-mRNA splicing^11–15^. Many studies have revealed that these NTP-dependent transcription activators play a critical role in abiotic stress response^16–23^. The expression of one of the potential stress responsive helicases, pea DNA helicase 45 (*PDH45*) is induced by salinity, dehydration, wounding and low temperature, suggesting it to be a general factor involved in abiotic stress adaptation^22^. Consistent with this, over expression of *PDH45* demonstrated enhanced abiotic stress tolerance^9, 18, 22, 24–27^ in several crops.

Chili (*Capsicum annum* L.), belonging to Solanaceae family is one of the important spice crops of the world. It is widely consumed as a vegetable and/or spice as the fruits are rich in health-enriching phytochemicals such as vitamins A, C, and B-complex and minerals^28^. Like other crops, economic harvest of chili is affected by a multitude of biotic and abiotic stresses^29–33^. Additionally, genetic transformation of chili is fastidious as it is recalcitrant to tissue culture regeneration^34^. In the present study, we demonstrate the wide functionality of a DNA helicase in transgenic chili under multiple abiotic stress conditions and exemplify the applicability of a tissue culture-independent apical meristem-targeted *in planta* transformation strategy for chili improvement.

## Results

### Development and selection of promising transgenic chili plants over-expressing *PDH45*

A total of 124 primary chili transformants (T_0_) were obtained by deploying the apical meristem-targeted *in planta* transformation strategy. Preliminary screening of T_1_ seedlings at 150 ppm kanamycin resulted in 700 seedlings that showed germination and proper root establishment (Supplementary Fig. S1a). Under non-stress conditions, these T_1_ transgenic plants were morphologically and phenotypically robust than the wild type (WT) and exhibited improved plant height, biomass and yield (Supplementary Fig. S1b). Molecular analysis using primers specific to the region spanning *PDH45* gene-CaMV35S promoter and *npt*II respectively confirmed the presence of T-DNA in the putative transformants (Supplementary Fig. S1c). Fruit yield in these plants ranged from 140–240 g/plant as compared to 148 g/plant in the wild type (WT) (Supplementary Fig. S1d). Fifty five PCR positive T_1_ plants with better growth, vigour and yield were advanced to T_2_ generation. PCR analysis of T_2_ plants demonstrated the stability of transgenes (Supplementary Fig. S2a-representative gel) in these plants. Fruit yield in the transgenic events was 120–280 g/plant as compared to 129 g/plant in the WT (Supplementary Fig. S2b). Following T_2_ analysis, five phenotypically robust and high yielding lines (16-1, 58-11, 41-5, 87-3 and 104-6) were advanced to T_3_ generation under greenhouse conditions.

**Figure 1:**
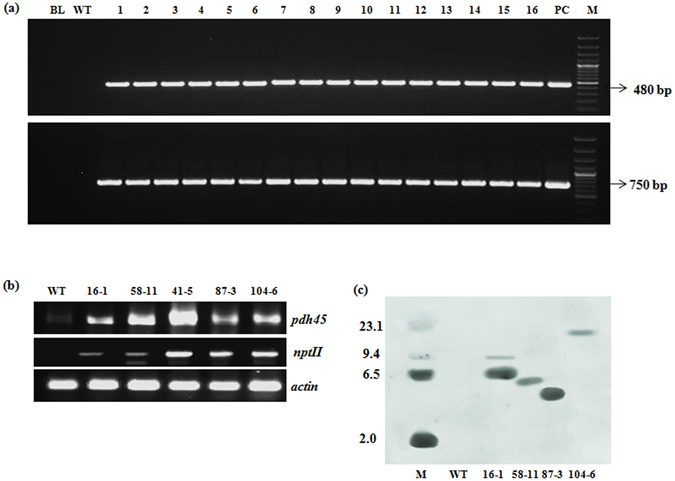
Molecular analysis of transgenic events in T_3_ generation. (**a**) PCR (BL = blank, WT = wild type, lane 1–16 = representative samples of putative transformants, PC = plasmid; M = 100 bp DNA marker- PCR and lamba *Hind*III digest-genomic Southern analysis); (**b**) sqRT-PCR; and (**c**) Genomic southern analysis of *Hind*III digested genomic DNA for ascertaining transgene (*npt*II) copy number in the transgenic events.

In T_3_ generation, PCR analysis of progeny of the selected events showed the expected amplicon of 480 bp with primers specific to CaMV*35S:PDH45* junction region and 750 bp specific to *npt*II gene (Fig. 1a-representative gel). sqRT-PCR analysis of five transgenic events further authenticated ectopic expression of *PDH45* (Fig. 1b) and *nptII* transgenes. Genomic Southern analysis with *npt*II as a probe demonstrated that the T-DNA was integrated and inherited either as single (transgenic events 58-11, 87-3 and 104-6) or double copies (transgenic event 16-1) (Fig. 1c). Stress-response experiments were carried out with the 5 selected events in T3 generation; seeds from the same subset were used for both seedling and plant level experiments.

### Evaluation of transgenic events for phenotypic traits under non-stress conditions

**Figure 2:**
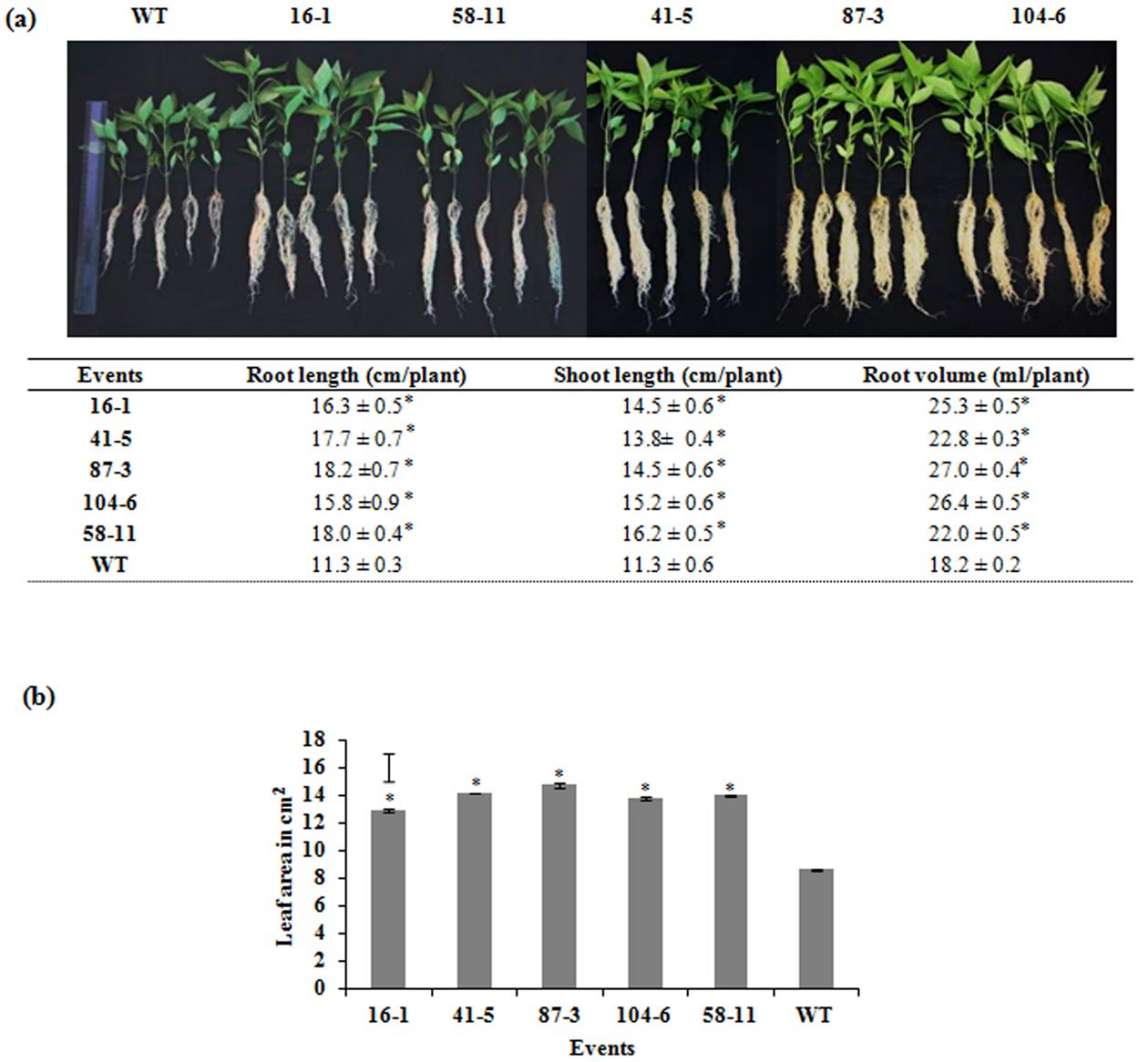
Morphometric evaluation of transgenic events under non-stress conditions. (**a**) Comparison of growth parameters (root length, shoot length and root volume) in the transgenic events (T_3_) and WT, 40 DAS under well-watered greenhouse conditions; (**b**) leaf area (cm^2^) in the transgenic events compared to WT. (Bars in the graph indicate LSD value at P < 0.05; (*) indicates significant differences at P < 0.05; (**) indicates significant differences at P < 0.01).

An attempt was made to evaluate the phenotype of the transgenic events in comparison to WT. Under non-stress conditions, 40 days old transgenic events showed significantly higher (P < 0.05) root length, shoot length and root volume compared to WT (Fig. 2a). The total leaf area of the transgenic events was also significantly higher in all the selected transgenic events illustrating the improvement in growth and carbon gain (Fig. 2b).

### Photosynthetic parameters and yield analysis in the transgenic events under non-stress conditions

Photosynthetic rate of the five selected transgenic events ranged from 26.7 to 31.4 µmol CO_2_ m^−2^ s^−1^ (Supplementary Fig. S3a) with transgenic events 87-3, 58-11 and 104-6 demonstrating improved carbon assimilatory capacity compared to WT (28.0 µmol CO_2_ m^−2^ s^−1^). Stomatal diffusive conductance (gs) varied among the five events (0.47–0.62 mol m^−2^ s^−1^) and was significantly higher in the events, 58-11 and 104-6 (Supplementary Fig. S3b). To delineate the roles of stomatal diffusivity and the chloroplast carboxylation efficiency, additional gas exchange parameters such as internal CO_2_ concentration (Supplementary Fig. S3c), transpiration rate (Supplementary Fig. S3d) and the ratio of intercellular CO_2_ concentration to stomatal conductance (Ci/gs) were compared (Supplementary Fig. S3e) which revealed an improved photosynthetic efficiency in the transgenic events over WT.

Under non-stress conditions, transgenic events recorded 25–58% yield advantage over WT plants (Supplementary Fig. S3f). Yield per plant in all the transgenic events (192–289 g/plant) was significantly higher (P < 0.05) than that of WT (154 g/plant) demonstrating improved yield due to better growth and photosynthesis.

### Cellular-level tolerance to various abiotic stresses under simulated conditions at seedling level

#### PEG6000-induced moisture stress

**Figure 3:**
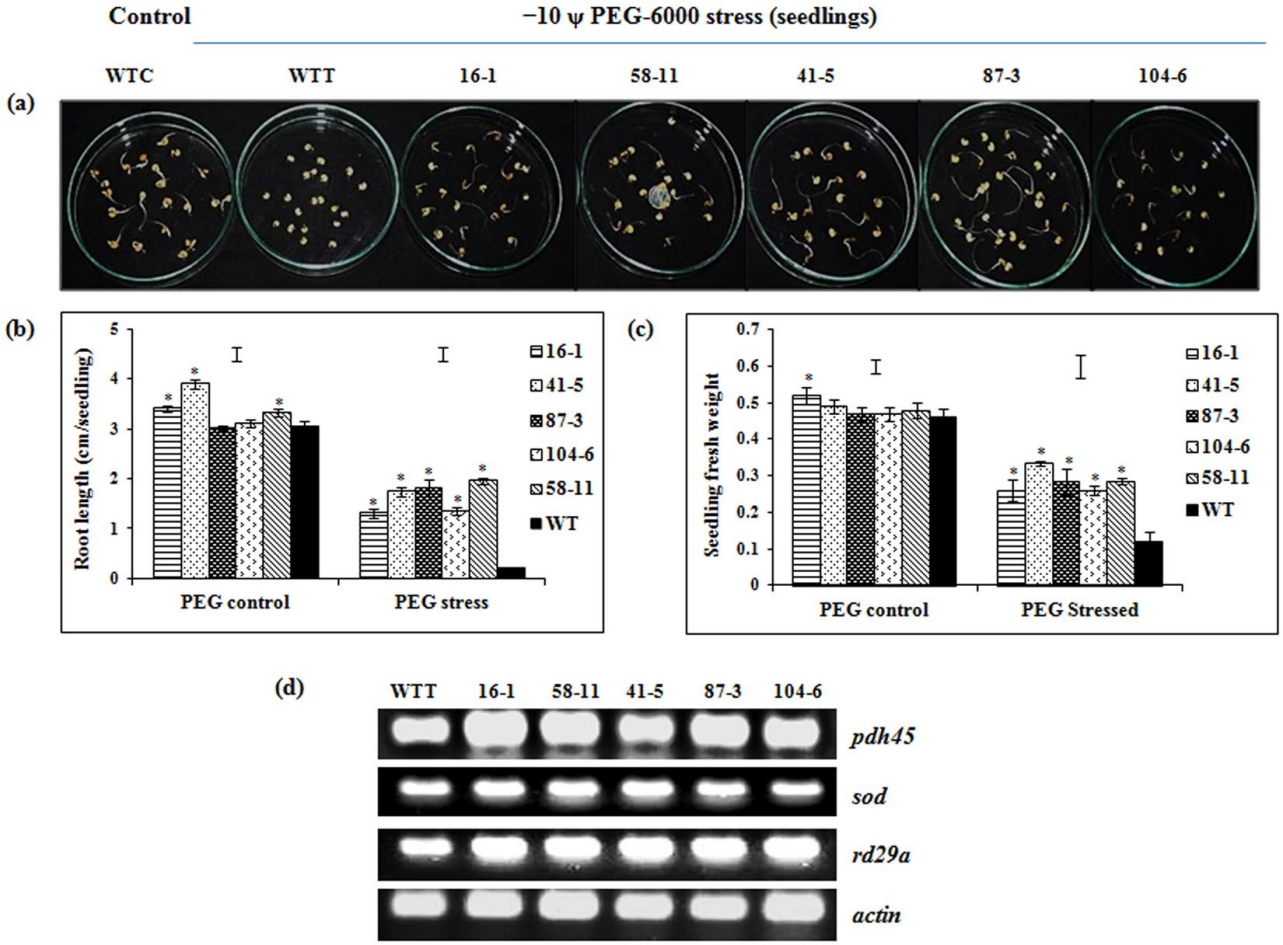
Response of the selected transgenic events to simulated moisture stress. (**a**) Effect of moisture stress (−10 ψ PEG-6000) on the seedlings of transgenic events and wild type; (**b**) seedling root length (cm); (**c**) seedling fresh weight (g); (**d**) sqRT-PCR analysis of *PDH45* and stress-responsive genes in the stressed seedlings. (WTC = wild type control; WTT = wild type treated. Bars in the graph indicate LSD value at P < 0.05; (*) indicates significant differences at P < 0.05.

Under simulated moisture stress using PEG6000 (Ψ = −10 bars), the transgenic events showed significantly higher root length (1.3–2.0 cm/seedling) (Fig. 3a and b) and seedling fresh weight (0.26–0.34 g/plate) (Fig. 3c) compared to WT (0.2 cm/seedling and 0.12 g/plant respectively). Additionally, sqRT-PCR revealed increased transcript accumulation of selected drought-responsive genes (*PDH45*, *SOD* and *RD29A*) in the transgenic events (Fig. 3d).

#### NaCl-induced salt stress

**Figure 4:**
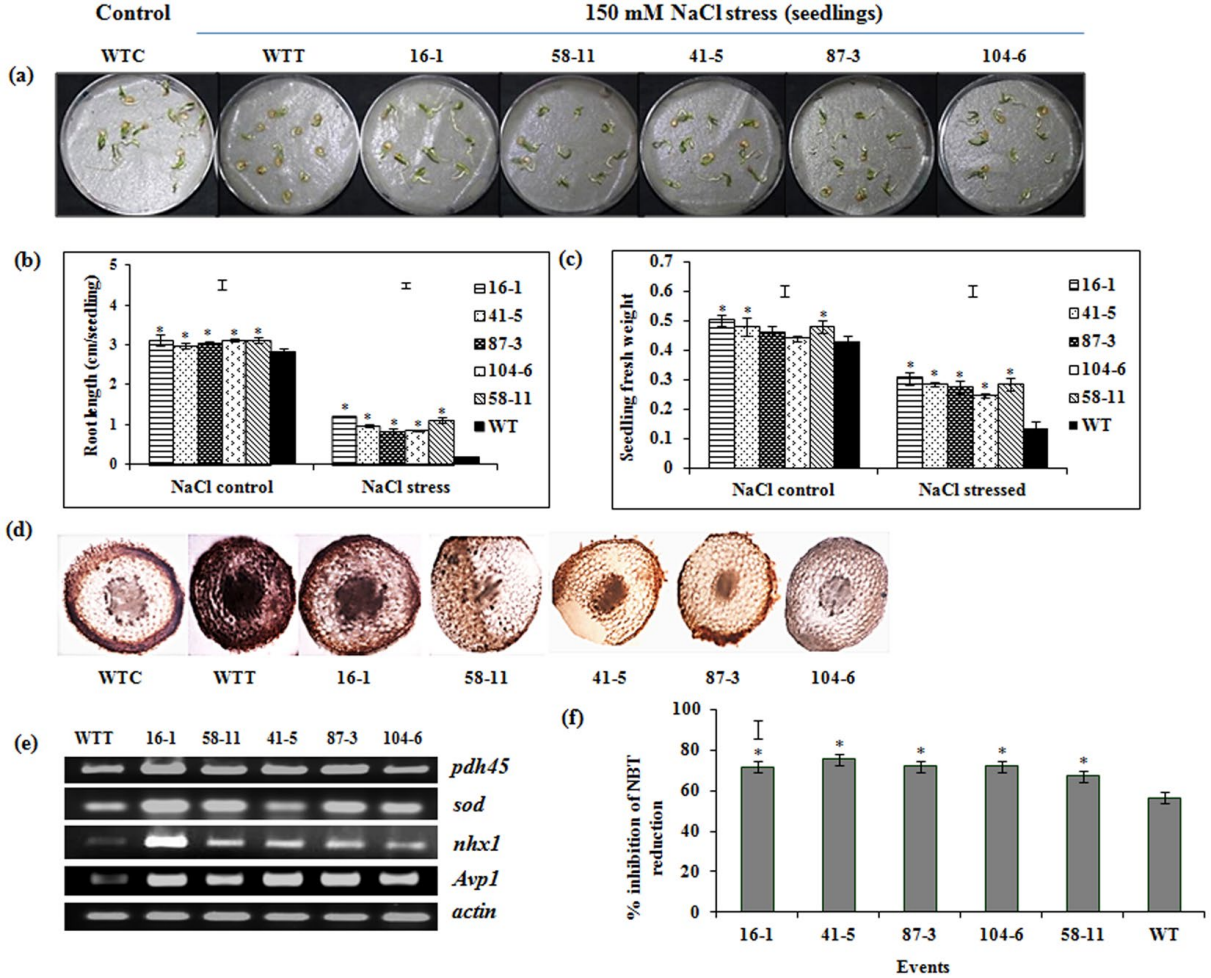
Response of the selected transgenic events to simulated salt stress. (**a**) Effect of salt stress (150 mM NaCl) on the seedlings of transgenic events and WT; (**b**) seedling root length (cm); (**c**) seedling fresh weight (g); (**d**) cross sections of roots stained with DAB; (**e**) sqRT-PCR analysis of *PDH45* and stress-responsive genes in the stressed seedlings; (**f**) SOD activity in terms of percent inhibition of NBT reduction in the seedlings of WT and transgenic events treated with 150 mM NaCl. (WTC = wild type control; WTT = wild type treated. Bars in the graph area indicate LSD value at P < 0.05; (*) indicates significant differences at P < 0.05.

In the presence of 150 mM NaCl (Fig. 4a), all the transgenic events recorded significantly higher root length (0.8–1.2 cm/seedling) and fresh weight (0.25–0.31 g/seedling) compared to WT (0.2 cm/seedling and 0.14 g/seedling respectively) (Fig. 4b and c). Root sections of these seedlings stained with DAB (Diamino benzidine) indicated that transgenic events 58-11, 41-5, 87-3 and 104-6 were the least accumulators of hydrogen peroxide (H_2_O_2_) compared to WT (Fig. 4d). sqRT-PCR analysis of *PDH45* and other salt stress-responsive genes such as superoxide dismutase, *SOD*; sodium–proton transporter, *NHX1*; Arabidopsis vacuolar H+-pyrophosphatase gene, *AVP1* showed higher transcript accumulation in all the transgenic events compared to WT (Fig. 4e). Concomitantly, significant (P < 0.05) increase in SOD activity (Fig. 4f) was observed in transgenic seedlings in terms of percent inhibition of NBT reduction (67–75%) indicating reduced salt stress-induced oxidative damage when compared to WT (56%).

#### Methyl viologen-induced oxidative stress

**Figure 5:**
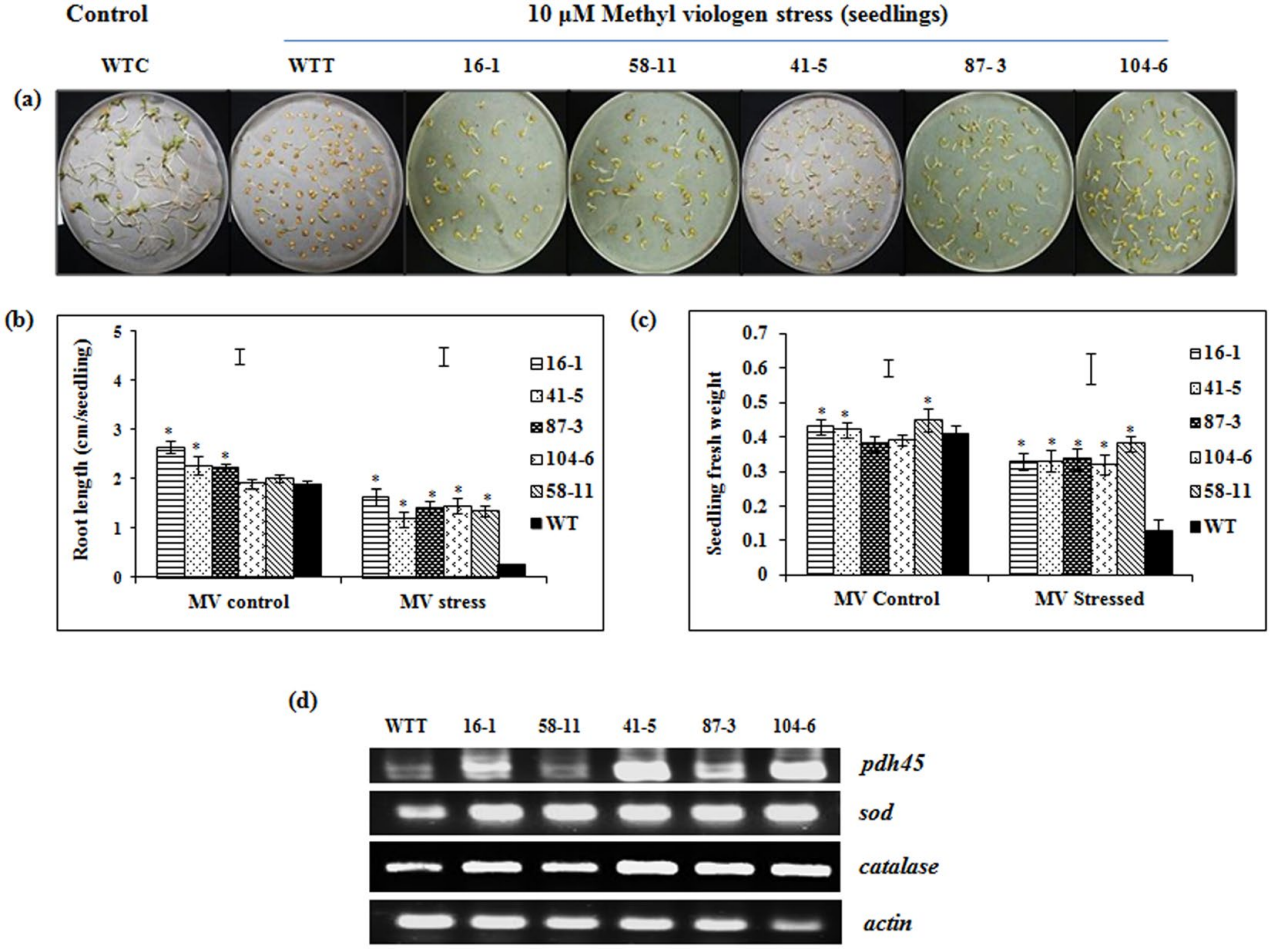
Response of the selected transgenic events to simulated oxidative stress induced by 10 µM methyl viologen. (**a**) Effect of oxidative stress on the seedlings of transgenic events and WT; (**b**) seedling root length (cm); (**c**) seedling fresh weight (g); (**d**) sqRT-PCR analysis of *PDH45* and stress-responsive genes in the stressed seedlings. (WTC = wild type control; WTT = wild type treated. Bars in the graph area indicate LSD value at P < 0.05; (*) indicates significant differences at P < 0.05.

Root length and seedling weight of the five selected transgenic events (1.2–1.6 cm/seedling and 0.32–0.38 g/plate respectively) under methyl viologen-induced oxidative stress was significantly higher than WT (0.26 cm/seedling and 0.13 g/plate) (Fig. 5a–c). Increased transcript accumulation of *SOD* was observed in the stressed seedlings of all transgenic events compared to WT (Fig. 5d), whereas expression of *PDH45* and *CATALASE* seemed to be slightly higher in the seedlings of transgenic events 41-5, 87-3 and 104-6 than WT as demonstrated by sqRT-PCR.

### Cellular-level tolerance in transgenic events under simulated abiotic stress conditions at plant level

#### Etheral-induced senescence stress

**Figure 6:**
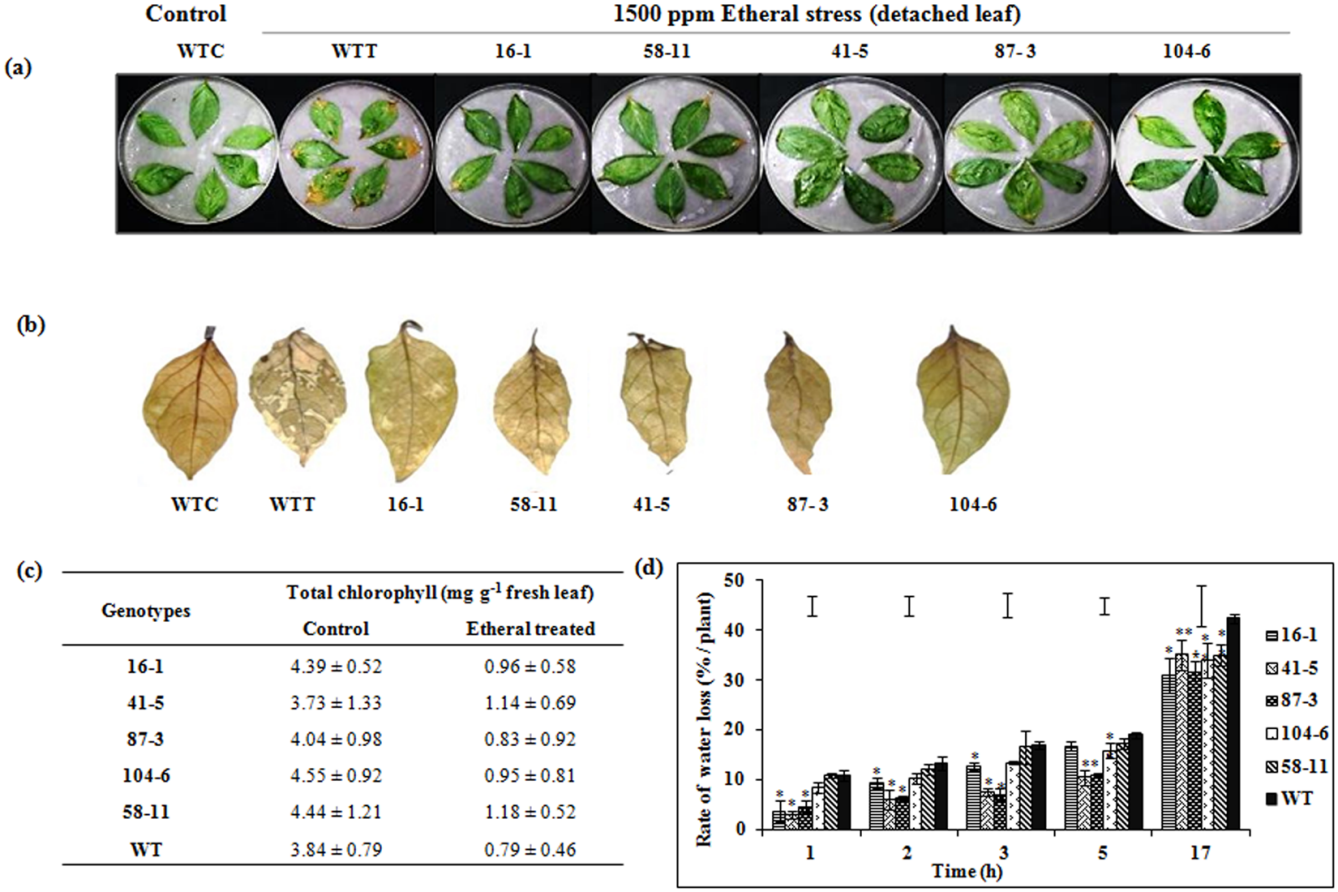
Response of the selected transgenic events to simulated stress at plant level. (**a**) Effect of 1500 ppm etheral on the detached leaves of selected transgenic events and WT; (**b**) DAB staining of leaves to assess the extent of tissue damage due to etheral stress; (**c**) total chlorophyll content measured in leaves under non-stressed and 1500 ppm etheral-induced senescence stress; (**d**) measurement of rate of water loss in the detached leaves of transgenic events and WT. (WTT - wild type treated, WTC - wild type control; Bars in the graph area indicate LSD value at P < 0.05; (*) indicates significant differences at P < 0.05; (**) indicates significant differences at P < 0.1).

The transgenic events not only showed reduced chlorosis but also retardation of senescence compared to WT as demonstrated by a detached leaf assay in the presence of 1500 ppm etheral (Fig. 6a). Further to stress imposition and DAB-staining, the leaves showed excessive tissue damage in WT than the transgenic events (Fig. 6b) indicating delayed senescence due to efficient chlorophyll stability in the transgenic events (Fig. 6c).

#### Rate of water loss

Based on a detached leaf assay, the study demonstrated reduced rate of water loss across various time points (1 h, 2 h, 3 h, 5 h and 17 h) in all the transgenic events (Fig. 6d) compared to the WT. However, transgenic events 41-5 and 58-11 showed promising response as they dehydrated at a slower pace compared to other events. Significant difference (P < 0.05) in the rate of water loss at the end of 17 h was observed and ranged between 30 and 33% in the transgenic events compared to 42% in the WT.

### Growth, WUE and yield in transgenic events under varied water stress levels

#### Water stress response of the transgenic events using a gravimetric approach

**Figure 7:**
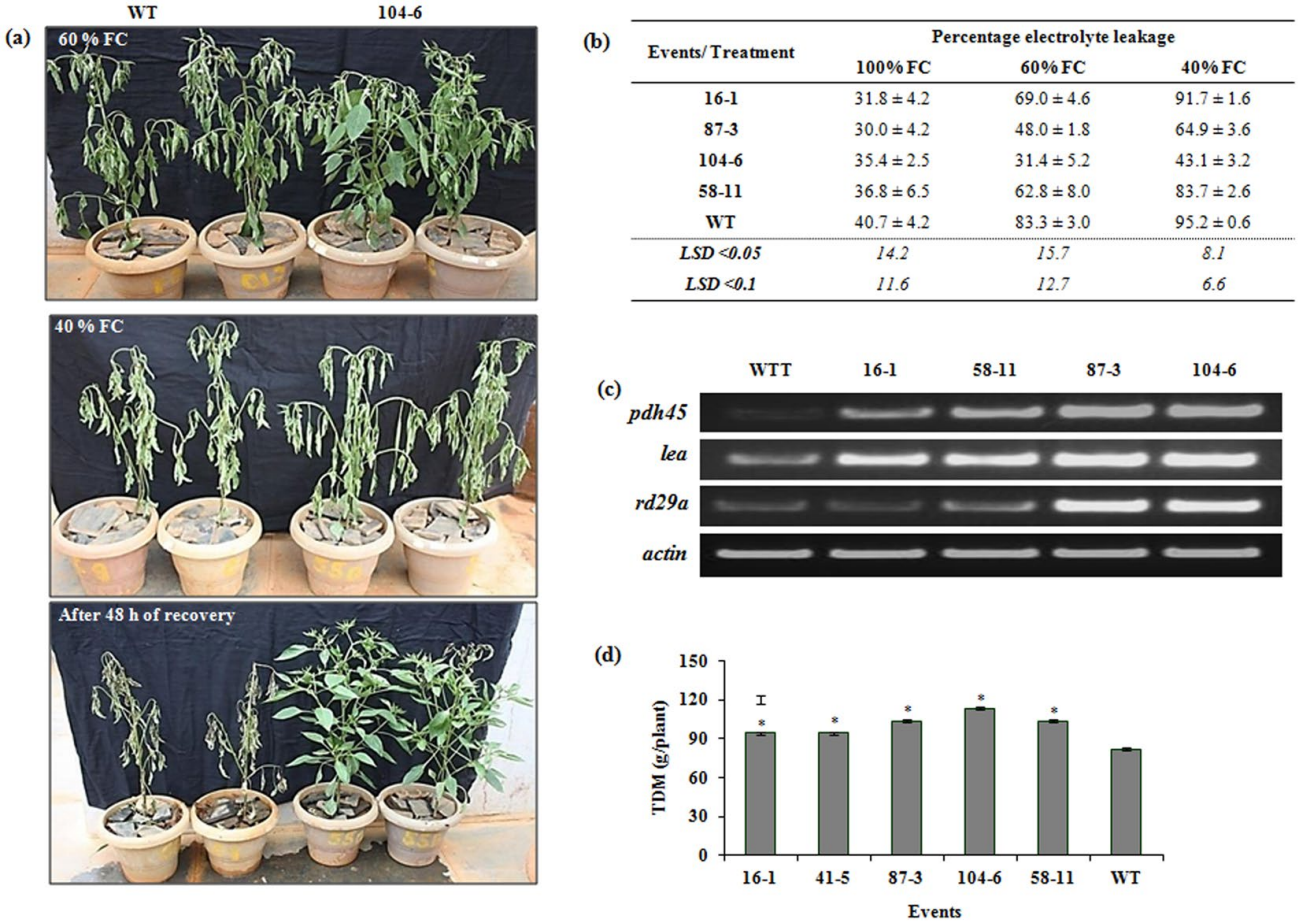
Response of the transgenic events to water stress at varied levels by gravimetric approach. (**a**) Drought tolerance in plants of WT and transgenic event 104-6 at 60% and 40% field capacity, and recovery response after 48 h of re-watering (100% FC); (**b**) membrane electrolyte leakage from different genotypes under well-watered and drought stress conditions; (**c**) expression of *PDH45* and other drought-responsive genes at 60% FC in different genotypes; (**d**) total dry matter (TDM) after recovery from drought stress in the transgenic events and WT. (WT = wild type; WTT = wild type treated. Bars in the graph area indicate LSD value at P < 0.05; (*) indicates significant differences at P < 0.05).

Gravimetric dry-down approach revealed conspicuous phenotypic differences between WT and transgenic event 104-6 (Fig. 7a) under different water stress levels (60% and 40% Field Capacity-FC). The transgenic event 104-6 showed partial wilting on par with WT plants at 60% FC; however, at 40% FC, WT plants exhibited severe wilting compared to 104-6 (Fig. 7a). Interestingly, when FC was restored to 100%, 104-6 completely revived within 48 h with turgid and healthy leaves compared to WT (Fig. 7a). However, leaf membrane integrity of all the five events exposed to progressive drying, showed significantly lower membrane leakage. At 60% and 40% FC, transgenic events 16-1 (69% and 91%), 58-11 (68% and 83%), 87-3 (48% and 64%) and 104-6 (31% and 43%) exhibited less membrane leakage compared to WT (83% and 95%) (Fig. 7b). Transgenic plants accumulated more transcripts of *PDH45* at 60% FC as revealed by sqRT-PCR (Fig. 7c). A similar pattern was also observed for *LEA* and *RD29A* with distinct increase in transcripts in the samples of transgenic events 87-3 and 104-6 (Fig. 7c). Improved growth under stress depicted by total dry matter (TDM) was evident in the transgenic events as they showed significantly higher (P < 0.05) (94–114 g/plant) TDM compared to WT (82 g/plant) (Fig. 7d) after 48 h of recovery.

#### Water use efficiency and productivity of transgenic events under moderate stress

**Figure 8:**
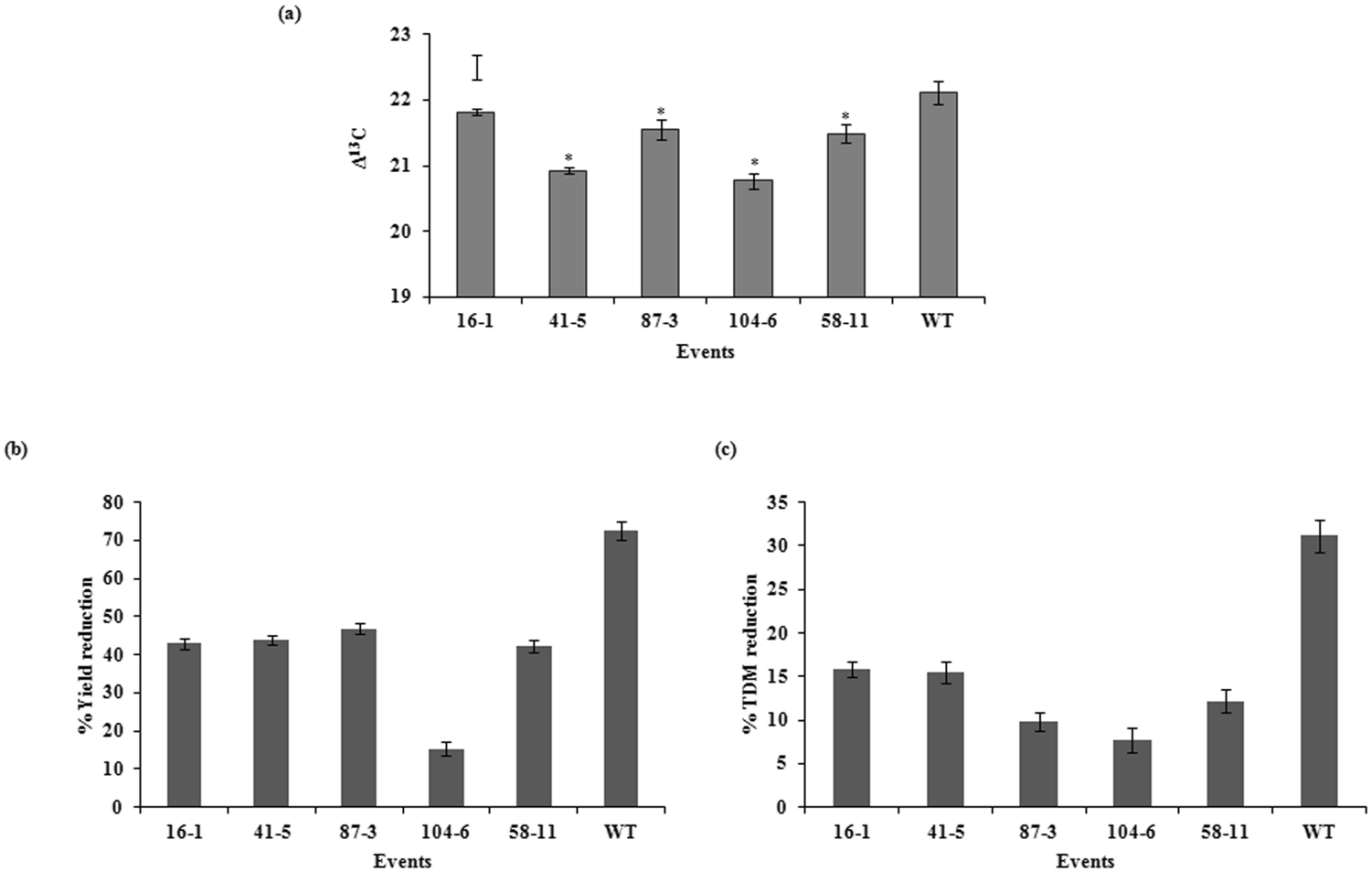
Water use efficiency and yield in the transgenic plants at moderate water stress of 70% FC. (**a**) Carbon isotope discrimination (Δ^13^C) in the leaves of transgenic events and WT; percent reduction in yield (**b**) and TDM (**c**) in the transgenic events at maturity when compared to WT.

The transgenic events exhibited improved growth, WUE and yield under moderate stress of 70% FC (Fig. 8). Carbon isotope discrimination (CID; ∆^13^C), a well established surrogate for WUE on a time integrated scale was used to compare the performance of transgenic events with the WT. It was observed that all the identified promising transgenic events showed lower ∆^13^C values when compared to wild type (Fig. 8a) indicating an increased WUE among the transgenic events. Impact of moderate stress was considerably less on productivity and biomass accumulation respectively of the transgenic events when compared to wild type (Fig. 8b and c). The transgenic events showed 18–45% reduction in fruit yield compared to 70% reduction in the wild type. The improved phenotype of transgenic events (as evident earlier in the study) was reproducible even under moderate stress as depicted by the reduced effect of stress on TDM (Fig. 8c) that exhibited 7–16% reduction *vis-à-vis* 30% reduction in wild type plants.

## Discussion

Although breeding for combinatorial stress tolerance is being attempted through deployment of marker assisted strategies, recent investigations have reported that introgression of a single gene into the genome of crop plants can also result in multiple stress tolerance^24^. Furthermore, transgenic technology through its ability to introduce genes from cross incompatible plant species is often considered as a precise technology for crop improvement. Among multiple single genes conferring abiotic stress tolerance, DNA and RNA helicases have proved their translational efficacy in multiple crops by improving tolerance to salinity and drought stress^9, 18, 22, 24–27^. DNA and RNA helicases, also known as molecular motors, are involved in myriad cellular processes of protein turnover and protection. Several mechanisms involving helicases in plant stress tolerance have been proposed^10^. Our earlier study in groundnut^18^ had unequivocally demonstrated the effect of ectopic expression of *PDH45* on improvement of cellular level tolerance. The present study in chili, a first of its kind, demonstrated the utility of *PDH45* to alleviate multiple abiotic stresses like salinity, oxidative stress, senescence and drought through transgenesis. This study therefore, is an explicit demonstration of the role of helicases in abiotic stress management in a vegetable spice.

The apical meristem-targeted *in planta* transformation coupled with stringent kanamycin selection was used to develop chili transformants^35^. Thorough evaluation in successive generations identified five homozygous T_3_ plants (16-1, 58-11, 41-5, 87-3 and 104-6) from 5 independent T_1_ events as promising. The role of *PDH45* in alleviating multiple abiotic stresses in chilli was assessed in these events following stress imposition.

All the five transgenic events expressing *PDH45* showed better growth under water limited and well-watered conditions as indicated by significantly higher above ground biomass. A considerable improvement in root traits such as root length and root volume leading to better maintenance of water relations was also observed in the transgenic events. Most of the transgenic events, especially 104-6, 58-11 and 87-3 revealed a higher stomatal conductance and photosynthetic rate under optimal irrigation conditions. The differences in photosynthesis are known to be driven predominantly by stomatal conductance, a turgor mediated phenomenon. Better root traits in the transgenic events might have led to higher leaf tissue water relations by maintenance of higher stomatal conductance thus leading to higher photosynthetic rates. A strong correlation between gs and photosynthetic rate per unit leaf area indicates a strong stomatal control of photosynthesis in these events. The ratio of assimilation rate to stomatal conductance (A/gs) (data not shown), which is often referred to as intrinsic water use efficiency at single leaf level, did not show significant difference and further reiterated the stomatal control of photosynthesis.

Due to the combined influence of acquired tolerance and maintenance of water relations, the transgenic events maintained much higher canopy leaf area (Fig. 2b). It is evident that with better carbon assimilation rate, these transgenic events eventually maintained higher canopy photosynthesis leading to higher total biomass. Although stomatal conductance regulates photosynthesis, a subtle contribution from a superior carboxylation efficiency may have also led to better maintenance of photosynthesis in the transgenic events. The ratio of intercellular CO_2_ concentration (Ci) to the stomatal conductance (gs), often considered as a rapid measure of intrinsic carboxylation efficiency^36, 37^, demonstrated an improvement in chloroplast capacity to fix carbon in the transgenic events compared to wild type. This was further proved by the lower carbon isotope discrimination values (Δ^13^C) in the transgenic events compared to wild type under moderate stress conditions (Fig. 8a). Stomatal conductance is also known to be positively related to ∆^13^C^38, 39^. In the present investigation, transgenic events 104-6 and 41-5 had significantly lower ∆^13^C despite a relatively high gs. This trend is possible when capacity of chloroplast to assimilate carbon is also enhanced. Further investigations would demonstrate which specific aspect of the chloroplast carbon assimilatory capacity is improved in the transgenic plants.

Being a transcription activator, involvement of *PDH45* in modulation of diverse biotic and abiotic cellular processes^10^ was conclusively demonstrated in the present study with efficient maintenance of growth under several simulated stress conditions. Further, the higher level of expression of stress responsive genes in transgenic chili demonstrated the role of *PDH45* in regulating expression of stress responsive genes. Stress-induced growth reduction is often associated with the inability to manage oxidative stress either by reduction in the production of ROS or by effectively scavenging them. A significantly higher SOD activity in transgenic events of chilli demonstrated their ability to scavenge ROS. Additional proof for reduced photo-oxidative damage was demonstrated by improved chlorophyll stability in the transgenic plants compared to wild type as evidenced either from reduced production of ROS or their effective scavenging. Maintenance of chlorophyll in etheral-induced stress condition is a sensitive assay for chlorophyll stability which in turn reflects mitigation of oxidative stress damage. The transgenic events exhibited higher chlorophyll content under etheral-induced senescence (Fig. 6a). Studies have demonstrated that relative chlorophyll content positively influences the photosynthetic rate^24, 40^ and improved chlorophyll stability or the stay green trait can be associated with improved yield under water-limited conditions^18, 24^. This evidence could be correlated to the improved yield observed in the transgenic plants of the present study (Supplementary Fig. S3f and Fig. 8b).

Our study also employed gravimetric approach to demonstrate the performance of the transgenic events under progressive water-limited conditions. It was observed that increased carbon assimilation per unit leaf area, maintenance of turgor by virtue of improved acquired tolerance to stresses enhanced the overall growth rate in the transgenic events in a concerted manner when compared to wild type. Therefore, it is plausible that the total biomass accumulation in the transgenic events both under well-watered and water limited conditions is governed by a significant increase in canopy photosynthesis. Because of the efficient ability to maintain membrane permeability and cell viability, the transgenic events showed a significant improvement in recovery growth upon stress alleviation. At 40% FC, 104-6 emerged to be the best performing (Fig. 7a) event with excellent post stress recovery. Preliminary studies showed the increased transcript accumulation of drought stress-related genes in the selected plants (Fig. 7c). Further investigations would provide insight on the global gene expression in the selected transgenic events under specific stress conditions^41, 42^.

Therefore, various simulated stress response studies demonstrated the potential of *PDH45* to improve cellular level tolerance against multiple abiotic stresses in chilli including improved growth and productivity. The study also emerged as an additional evidence that demonstrated the utility of *PDH45* to combat abiotic stresses. Though similar cellular level tolerance by helicases has been demonstrated, the present study is the first evidence that delineates coherence in alleviating multiple stresses by a single gene. The results directly indicate that there exists an intrinsic crosstalk between various stresses. These positive responses to various stresses indicate that helicase may be able to impart tolerance by acting at the translation level in the removal of secondary structures and thereby enhancing protein synthesis. The success of these *in vitro* experiments could pave way for field level experiments essential to assess the tolerance of transgenic plants to various natural vagaries.

## Methods

### Development and selection of promising chili transgenic events overexpressing *35 S::PDH45*

#### Vector for plant transformation

The cDNA of the complete open reading frame of *PDH45* gene (1.2 kb) was cloned into the binary vector pBI121 under the control of CaMV*35S* promoter and *nos* terminator at the *Xba*I site. The binary vector harbouring the transgene (*PDH45*) and the selectable marker *npt*II, conferring kanamycin resistance, was mobilized into the disarmed *Agrobacterium tumefaciens* strain EHA105^43^ and used for transformation.

#### Plant material and in planta transformation

Five day-old germinated seedlings of chili (cultivar *fof-2*) were used as explants for *in planta* transformation^35, 44^ using *Agrobacterium tumefaciens* containing *PDH45*. T_1_ seeds from primary transformants were harvested and screened for the selection of putative transformants.

#### Screening for the selection of putative transformants

A minimum inhibitory concentration (MIC) of 150 ppm kanamycin was used for screening T_1_ transformants. Four day old germinating seedlings were soaked in 150 ppm kanamycin for 2 h at room temperature and transferred to a tray containing quartz sand. These seedlings were grown under greenhouse conditions for 10 days and supplemented with ¼^th^ strength Hoagland solution^45^. Well-established chili plants were transferred to pots and were analysed further for transgene integration.

#### Molecular analysis of transformants

*PCR analysis*. Total genomic DNA was isolated from leaves of both wild type and transgenic plants^46^ and quantified using Nanodrop®. The transgenic plants were analysed for the presence of *npt*II and the junction region between CaMV*35S* promoter and *PDH45* transgene using specific primers (Supplementary Table S1). The PCR program comprised of initial denaturation of 95 °C for 5 min followed by 35 cycles of 95 °C for 1 min, 58 °C for 1 min, 72 °C for 1 min, and final extension of 10 min at 72 °C. Each PCR reaction consisted of 100 ng of genomic DNA, 5 µl of 10X PCR buffer, 10 pM each of forward and reverse primer, 200 µM dNTPs, 1 U of Taq DNA polymerase and made up to a final volume of 50 µl with nuclease-free water. While “Blank” had nuclease-free water, negative control-2 contained 100 ng of genomic DNA of WT and positive control contained 25 ng of *pBI121:CaMV35S::PDH45* plasmid.

*Genomic Southern analysis for determination of T-DNA copy number*. Total genomic DNA (15 µg) was digested with *Hind*III (5U), electrophoresed on 0.8% agarose gel and transferred onto nitrocellulose membrane. Using the protocol suggested by Mega Prime DNA labelling kit (Amersham Pharmacia Biotech, UK), the 750 bp *npt*II DNA fragment was labelled with α[^32^P]-dCTP and hybridized to the target DNA for 18 h at 65 °C. The membrane was sequentially washed at 65 °C with wash buffer 1 (3X SSC containing 0.1% SDS), wash buffer 2 (0.5X SSC containing 0.1% SDS), and wash buffer 3 (0.1X SSC containing 0.1% SDS) for 30 min each^47^. The membrane was wrapped and exposed to an X-ray film in dark. After 36 h of exposure, the film was developed using Kodak developer and fixer.

*Expression analysis by sqRT-PCR*. Total RNA was isolated^48^ from leaves (both stressed and non-stressed plants) and stressed seedlings. Following quantification, RNA from transgenic and wild type plants was used for cDNA synthesis using oligo (dT) primers and Molonyl-Murine Leukaemia Virus reverse transcriptase (MMLV-RT) according to manufacturer’s instructions. sqRT-PCR reactions were setup using specific primers (Supplementary Table S2). PCR program of 3 min initial denaturation at 95 °C followed by 25 cycles of 95 °C for 1 min, 52–58 °C for 30 sec, 72 °C for 1 min, and a final extension of 10 min at 72 °C was used for ascertaining the variations in gene expression among the transgenic events *vis-à-vis* the WT.

### Morphometric evaluation of transgenic events under non-stress conditions

Separate experiments (as listed below) were conducted to analyse growth, photosynthetic ability and yield attributes in the transgenic plants under non-stress conditions.

**Experiment 1:** Five day-old germinated seedlings of T_1_ transgenic plants were transplanted into a tray containing quartz sand and allowed to grow under greenhouse conditions. These plants were supplemented with ¼^th^ strength of Hoagland solution^45^. Plants were carefully up rooted 40 DAS and were washed gently with water. Growth parameters in terms of shoot length, root volume and leaf area^49^ were measured.

**Experiment 2:** Photosynthetic efficiency was measured in the transformants and wild type plants that were raised in pots under greenhouse conditions. Gas exchange parameters were recorded from the middle portion of fully expanded third leaf of healthy 30–40 days old plants. Gas exchange in leaves was measured in ambient CO_2_ concentration (390 ppm). Measurements were recorded between 07:00 am and 11:00 am using a portable photosynthesis system (LI-6400, LI-COR, Lincolin, Nebraska, USA). The automated leaf chamber was fitted with facilities to provide light intensity between 0 and 3000 μmolm^−2^ s^−1^ through the red/blue LEDs, and peltier electronic coolers to maintain the temperature. The selected leaf was placed in the leaf chamber till the values stabilized and there after the gas exchange values were recorded. The leaf chambers were maintained close to ambient conditions to record realistic values. Various parameters such as photosynthetic rate (A), transpiration (T), stomatal conductance (gs) and internal CO_2_ concentration (Ci) were recorded^50^.

Further, the plants were grown till maturity and yield attributes in terms of total yield and total dry matter was recorded both in the transgenic events and wild type plants for comparative analysis.

### Evaluation of response of transgenic events under simulated stress conditions at seedling level

Uniformly germinated five-day old seedlings (15 seedlings per plate) of both WT and transgenic events were transferred to a Petri plate for stress imposition assays. Moisture stress was imposed by exposing the seedlings to −10ψ PEG6000 solution whereas salinity stress was imposed by exposing the seedlings to 150 mM NaCl; oxidative stress was simulated by exposing the seedlings to 10 µM methyl viologen. Following the simulated stress given separately for 48 h at 28 °C, the seedlings were transferred onto a fresh Petri plate containing wet blotting paper (with sterile distilled water) and incubated for 72 h at 28 °C for recovery. A set of WT seedlings grown in sterile distilled water was maintained as absolute control. Length and weight of the seedlings was measured to assess the effect of stress on seedling growth. Accumulation of hydrogen peroxide (H_2_O_2_) in the seedlings^51, 52^ as a measure of salt stress was estimated by measuring SOD activity using the photochemical NBT reduction^53^ and DAB staining of the roots.

### Evaluation of transgenic events under simulated stress conditions at plant level

#### Etheral-induced senescence

Well expanded (third leaf from the top) leaves of 35 days old plants were transferred to a Petri plate supplemented with 1500 ppm etheral and incubated in dark for 48 h at room temperature. The treated leaf was used for studying accumulation of endogenous H_2_O_2_ and subsequent loss of chlorophyll in the leaves using DAB staining^51, 52^. Another set of etheral-treated leaf discs was transferred to a test tube containing 10 ml of 80% acetone: di-methyl sulfoxide (DMSO) (1:1 v/v) solution and incubated in dark for 72 h. Chlorophyll *a* and *b* that leached out of the discs were measured spectrophotometrically at 665 nm and 650 nm, respectively^54^.

#### Rate of water loss

Concomitantly, transpirational water loss in plants was measured by a detached-leaf assay of the excised third leaf from wild type and transgenic chili plants^55^. Fresh weight of the leaves was recorded immediately after excision and further incubated in a Petri plate at 28 °C. The weight of these leaves was recorded after 1 h, 2 h, 3 h, 5 h and 17 h. The rate of water loss was calculated using the formula:

$${\rm{W}}{\rm{a}}{\rm{t}}{\rm{e}}{\rm{r}}\,{\rm{l}}{\rm{o}}{\rm{s}}{\rm{s}}\,({\rm{ \% }})=({\rm{f}}{\rm{r}}{\rm{e}}{\rm{s}}{\rm{h}}\,{\rm{w}}{\rm{e}}{\rm{i}}{\rm{g}}{\rm{h}}{\rm{t}}-{\rm{w}}{\rm{e}}{\rm{i}}{\rm{g}}{\rm{h}}{\rm{t}}\,{\rm{a}}{\rm{t}}\,{\rm{t}}{\rm{i}}{\rm{m}}{\rm{e}}\,{\rm{p}}{\rm{o}}{\rm{i}}{\rm{n}}{\rm{t}})/100.$$

### Evaluation of transgenic events to water stress under varied levels

#### Experiment 1: Analysis of water stress response by gravimetric approach

Transgenic events and WT plants were grown in 9 inch pots containing red soil under greenhouse conditions. At 35 DAS, the pots were saturated with water and maintained for five days at 100% field capacity (FC). Following this, the plants were subjected to progressive water stress imposed by gravimetric method^56^. Plants were first exposed to 60% FC for 5 days and then to 40% FC for 5 days. Leaves were sampled from the plants at 60% FC to analyse expression of a few drought responsive genes and both the stress regimes for membrane integrity. Recovery response of these plants was analysed by re-watering the pots to 100% FC. The plants were later harvested and total dry matter (TDM) was recorded.

Membrane damage in leaves was assessed by performing electrolyte leakage assay^56, 57^. Leaf discs were excised from the plants exposed to 100%, 60% and 40% FC and incubated for 2 h in deionized water. Initial electrical conductivity (EC) was recorded using EC-TDS analyser and the samples were then incubated for 15 min in a boiling water bath to measure the final EC. Electrolyte leakage of each sample was calculated using the formula, EC (%) = (initial EC/final EC) × 100.

#### Experiment 2: Evaluation of transgenic events for water use efficiency and productivity under moderate water stress

Initially, transgenic events and wild type plants were grown under greenhouse conditions for 35–40 days at 100% FC and moderate stress (70% FC) was imposed till maturity. WUE was measured by carbon isotope discrimination (CID) (Δ^13^C) using an Isotope Ratio Mass Spectrometer (IRMS). For this, mature and fully expanded leaves from the stressed transgenic and wild type plants were dried completely, powdered and used to determine Δ^13^C. At maturity, both transgenic and wild type plants were harvested and total yield and dry matter (TDM) were recorded.

### Statistical analysis

Analysis in all the experiments was carried out in triplicates. ANOVA was performed using GenStat 10.1.0.72 (VSN International Ltd., UK) to calculate the least significant difference (LSD) between mean values within treatments at 0.05 and 0.01 significance levels.

## Electronic supplementary material

Supplementary information
