# Supplementary material for: Overexpression of Pea DNA Helicase 45 (PDH45) imparts tolerance to multiple abiotic stresses in chili (Capsicum annuum L.)
Source: Sci Rep. 2017 Jun 5;7:2760. doi: 10.1038/s41598-017-02589-0 (PMC5459802; doi:10.1038/s41598-017-02589-0)
Supplement: Supplementary file 1 — Supplementary information [file 41598_2017_2589_MOESM1_ESM.pdf]

Overexpression of Pea DNA Helicase 45 (*PDH45*) imparts tolerance to multiple abiotic stresses in chili (*Capsicum annuum* L.)

Tagginahalli N. Shivakumara[1,2#], Rohini Sreevathsa[1,3#*], Prasanta K Dash[3], M.S Sheshshayee[1], Pradeep K. Papolu[2], Uma Rao[2], Narendra Tuteja[4], M. Udaya Kumar[1*]

Supplementary table S1: Primers used for PCR analysis

Name	Sequence (5' --- 3')
CaMV 35S F	GAAGGTGGCTCCTACAAATG
PDH45 R	CTATCATTCAGGGACGTGATG
npt II F	GAGGCT ATTCGGCTATGACTG
npt II R	ATCGCGAGG GGC GATACC GTA

Supplementary table S2: Primers used for sqRT-PCR analysis

Name	Sequence (5' --- 3')
SOD F	CCAGAAGATGAGCCCGCCATGC
SOD R	AGCGTTTCCGGTGGTCTTCGTC
NHX1 F	AATGCGCTCCAGAACTTTGA
NHX1 R	TCCCGCCAGAACTAATCCTA
AVP1 F	AGGAACCGCAAAACCACACTACG
AVP1 R	GCGAACAAGGGAGCAAAGACAAG
LEA F	CAGACAACACTCACCGATAGCA
LEA R	GCAGGAGATACAACAAGCAGATAA
CATALASE F	ACACTCAGAGGCACCGTCTT
CATALASE R	GCTTTCCGGACAACCTAACA
Rd29 F	TATAAAATAAAAGATCATACCTA
Rd29 R	CATTTTCCAAAGATTTTT
PDH45 F	ATGGCGACAACTTCTGTGG
PDH45 R	CCCAGGAGTTCCAGACACC

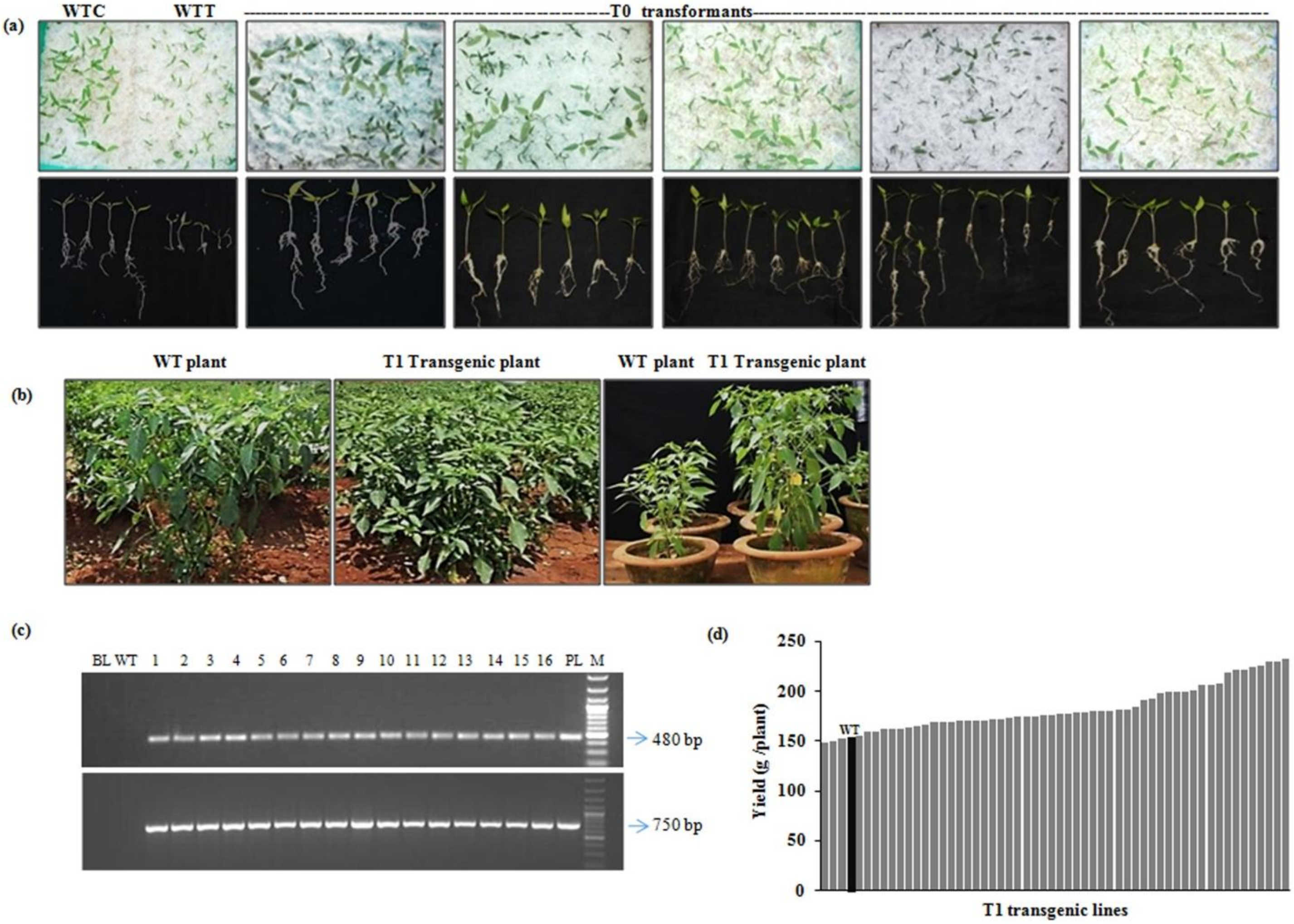

Supplementary figure S1: Analysis of T_1 generation plants

(a) Screening for the selection of putative transformants grown in sand system under selection pressure of 150 ppm kanamycin (Legend: WTC=wild type control under 0 mM kanamycin; WTT=wild type treated); (b) Comparison of phenotype of representative WT and transgenic plants grown in the greenhouse; (c) PCR analysis of the T_1 generation plants amplifying a 480 bp fragment (*35S:PDH45* junction region) and 750 bp *nptII* gene fragment (Legend: BL=blank, WT=wild type, lane 1–16=representative samples of putative transformants, PL=plasmid; M=100 bp DNA marker); (d) Fruit yield (dry weight) in T_1 generation plants grown under well-watered conditions in the greenhouse.

(a)

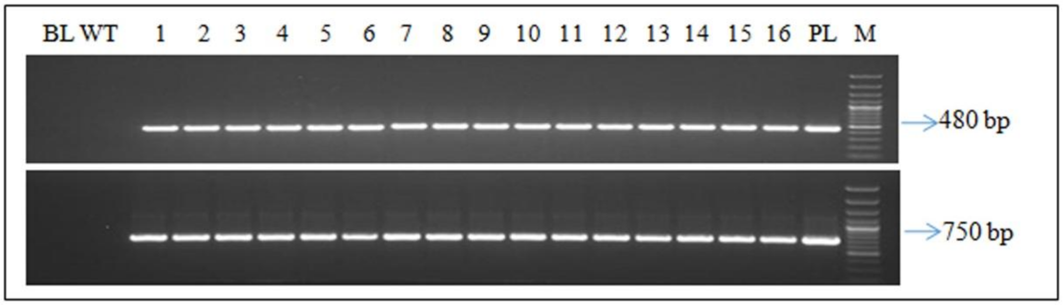

(b)

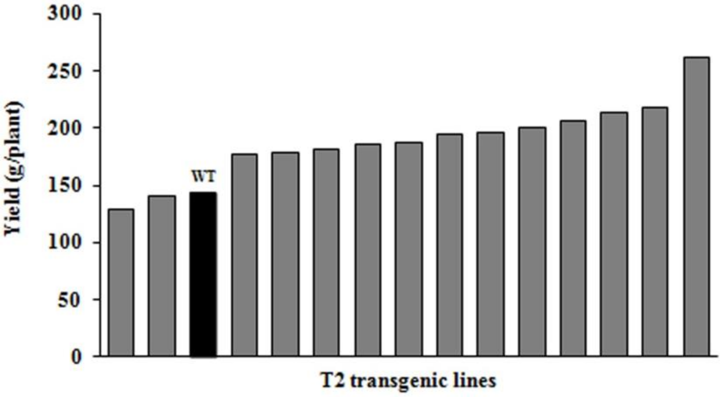

Supplementary figure S2: Analysis of T_2 generation plants

(a)PCR analysis of the T_2 generation plants amplifying a 480 bp fragment (*35S:PDH45* junction region) and 750 bp *nptII* gene (Legend: BL=blank, WT=wild typel, lane 1–16=representative samples of T_2 generation, PL=plasmid; M=100 bp DNA marker); (b) Yield in the selected T_2 generation plants.

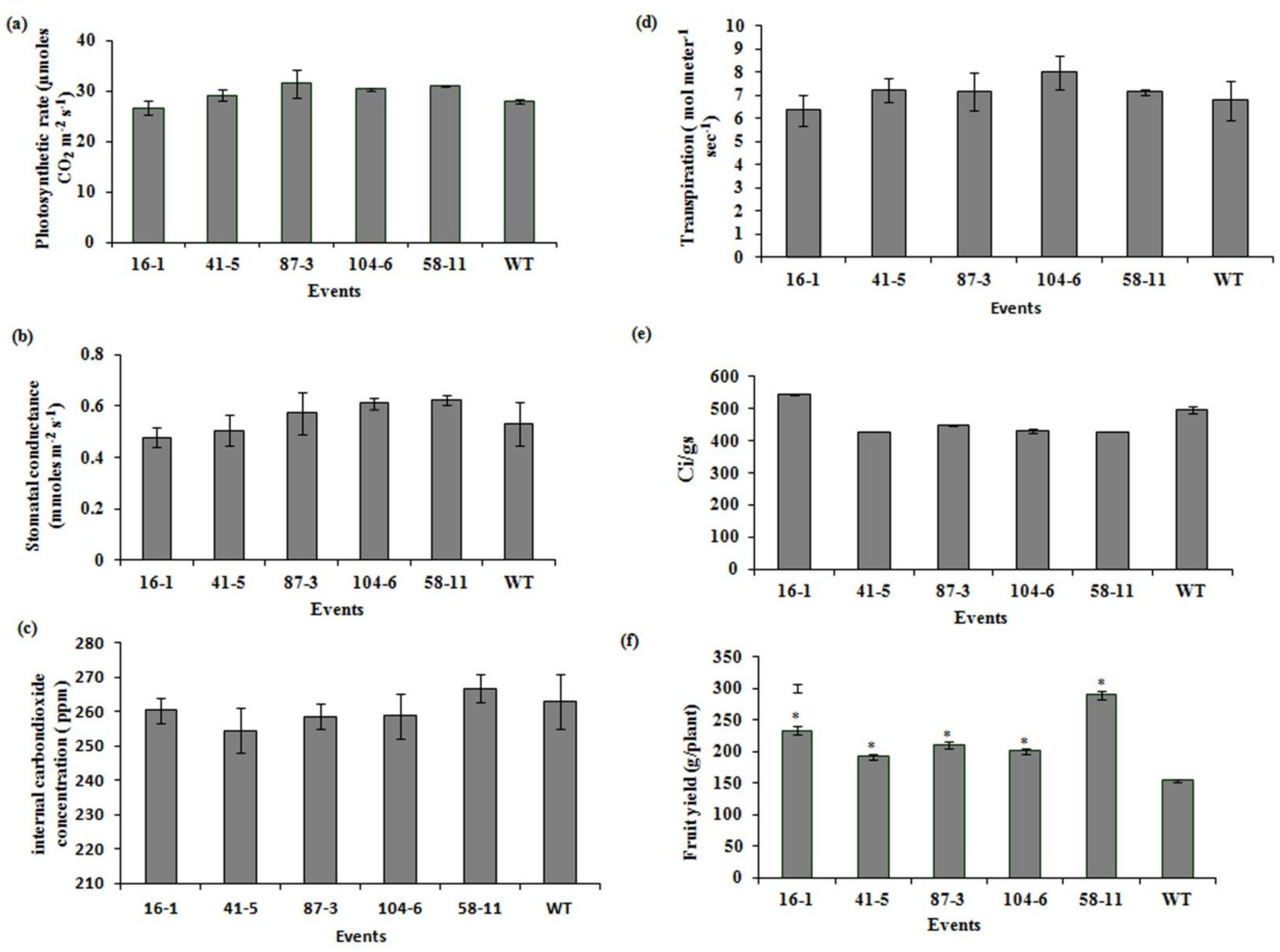

Supplementary Figure S3: (a-e) Gas exchange parameters in the transgenic events and wild type plants under non stress conditions. (f) Fruit yield (dry weight) obtained from the transgenic events grown under well-watered conditions in controlled environment

(Legend: Bars in the graph area indicate LSD value at $P<0.05$; Asterisk '*' indicates significant differences at $P<0.05$)
